# Data in support of dual-functionalized cellulose nanofibrils prepared through TEMPO-mediated oxidation and surface-initiated ATRP

**DOI:** 10.1016/j.dib.2015.03.003

**Published:** 2015-03-28

**Authors:** Tzung-Yung Tsai, Chih-Feng Huang

**Affiliations:** Department of Chemical Engineering, National Chung Hsing University, 250 Kuo Kuang Road, Taichung 402, Taiwan

## Abstract

We previously studied a suitably 2,2,6,6-tetramethylpiperidine-1-oxyl (TEMPO)-oxidized cellulose nanofibrils (TOCNs) that can be further functionalized with initiating sites and overcame the obstacle of performing atom transfer radical polymerization (ATRP) in the presence of neutral carboxylic acid sodium salt groups [1]. Herein, characterization of the modified TOCNs and of the products from surface-initiated (SI) ATRP of the (nano)celluloses with styrene (St) was performed using nuclear magnetic resonance (NMR), gel permeation chromatography (GPC), and contact angle (CA) measurements. From the analysis of ^1^H NMR, a high purity of sacrificial initiator (i.e., 2-hydroxyethyl 2-bromoisobutyrate (HEBiB)) was confirmed. HEBiB was utilized to trace the SI ATRP with the generated free PSt. Gradually molecular weight evaluations were revealed from GPC analysis (ca. *M*_n_=21,000 and *Đ*=1.10) using different TOCNs, implying the insignificant contribution to the kinetics from the grafted initiating sites. The TOCN-*g*-PSts were further characterized by contact angles and displayed an obvious reversibility between hydrophilicity and hydrophobicity in tens of minutes. These results illustrated a simple and facile approach for controlling the graft length and composition of TOCNs through SI ATRP.

Specifications table

**Table:** 

Subject area	Material science
More specific subject area	Surfaced-initiated atom transfer radical polymerization from nanocelluloses.
Type of data	Images (contact angles), figures (GPC, NMR).
How data was acquired	GPC: gel permeation chromatography (GPC) was performed in THF at a flow rate of 1 mL/min at 40 °C, employing a Waters 515 pump, a Waters 410 differential refractometer (RI), a Waters 486 absorbance detector (UV), and two PSS SDV columns (Linear S and 100 Å pore size) to estimate *M*_n_, *M*_w_, and *Đ* (i.e., *M*_w_/*M*_n_).
	Contact angles: KRÜSS G10 system.
Data format	Analyzed.
Experimental factors	Dried samples were pressed at ca. 50 psi to obtain flat platelets under ambient and dried in vacuum prior to measure CAs.
Experimental features	GPC: Monodisperse polystyrene standards were used for calibration. We monitored the MW of grafted PSt via the free PSt chains in solution.
Data source location	National Chung Hsing University, Taichung, Taiwan.
Data accessibility	Data is available with this article.

Value of the data

• A sacrificial initiator with high purity to monitor the SI ATRP kinetics was characterized.
• Contact angle measurements for comprehensive of surface images were shown.
• A living/controlled fashion using surface-initiated atom transfer radical polymerization was displayed.
• The data presented here can be used to characterize the tailor-made surface grafted polymer in length, length distributions, and compositions.

## 1 Data, experimental design, materials and methods [1]

### 1.1 Materials

Styrene (St, 99%) was purchased from Sigma-Aldrich and purified by passing through a column filled with basic alumina to remove the inhibitors or antioxidants. Bleached hard wood pulp was provided by Chung Hua Pulp through sulfate process treatment (LBKP grade). TEMPO (98%+), 2-bromoisobutyryl bromide (BiB, 97%), 4,4-dimethylaminopyridine (DMAP, 99%), and copper(I) bromide (CuBr, 98%) were purchased from Alfa Aesar. *N*,*N*,*N*´,*N*´´,*N*´´-Pentamethyldiethylenetriamine (PMDETA, 99%) and triethylamine (TEA, 99.5%) were purchased from Sigma-Aldrich. NaOH (96%), KBr (99%), HCl (35%), ethylene glycol (>99%), NaBr (99.5%), and NaClO_(aq)_ (12%) were purchased from Showa. CuBr was purified by washing with glacial acetic acid (to remove any soluble oxidized species), filtered, washed with EtOH, and dried under vacuum. All other solvents were distilled prior to use.

### 1.2 Preparations of TEMPO-oxidized cellulose nanofibrils (TOCNs)

TEMPO (0.10 g, 0.64 mmol) and NaBr (0.80 g, 7.8 mmol) were added to wood pulp (8.0 g) dispersed in deionized water (DI water, 600 mL). 1 M NaOH was added slowly until the mixture achieved pH 10, and then NaClO (80 g, 1.08 mol) was added to begin the oxidation/reduction reactions. After a desired time, the reaction was stopped by diluting with copious DI water. The product was purified by repeating the DI water washing/centrifugation cycle a few times, until the aqueous solution reached neutral pH. The solution was concentrated and a hydrogel was obtained (5 wt% of TOCN solid content; yield: 94.5%).

### 1.3 Surface modification of TOCN with ATRP initiating moiety (TOCN-Br)

To obtain a well-dispersed TOCN/DMF mixture, solvent exchange from TOCN/H_2_O to TOCN/DMF was conducted by sequential washing steps acetone–DMF extractions for three times using a hydrophilic polytetrafluoroethylene membrane filter (H010A090C, Toyo Roshi Kaisha, Ltd.). A mixture of TOCN (0.18 g) and DMF (45 mL) was sonicated in a round-bottom flask for 15 min using an ultrasonic homogenizer (200 W), and then 4,4-dimethylaminopyridine (DMAP) was added. Desired amounts of triethylamine (TEA) and 2-bromoisobutyryl bromide (BiB) were added dropwise into the mixture through an addition funnel. Reaction compositions and temperatures are summarized in Table 1. After the reaction was complete, saturated NH_4_OH_(aq)_ (40 mL) was added to stop the reaction; the product was washed through two cycles of Soxhlet extraction, with CH_2_Cl_2_ and MeOH, for 48 h. The product (i.e., TOCN-Br) was dried and stored in a desiccator (yield: 84.1%).

**Table 1:** Conditions for preparation of TOCNs and EA characterization data.

Sample^a^	BiB (mmol)	*t* (h)	C (%)	H (%)	O (%)	Br (%)^b^
TOCN	–	–	39.09	5.81	55.03	–
TOCN-Br1	80	24	43.61	5.64	46.17	4.58
TOCN-Br2	160	24	43.12	5.97	43.89	7.02
TOCN-Br3	160	48	48.12	5.69	37.49	8.70

a Reactions were performed at 40 °C in DMAc with TOCN (0.4 g), DMAP (0.2 g); TEA was added equal molar amounts with BiB.

b Estimated by assuming C (%)+H (%)+O (%)+Br (%)=100 (%).

### 1.4 Synthesis of 2-hydroxyethyl 2-bromoisobutyrate (HEBiB) [2], [3]

Anhydrous ethylene glycol (270 mL, 4.84 mol) and TEA (13 mL, 95 mmol) were mixed with dry DCM 100 mL in a three-neck 500 mL flask under nitrogen. 2-Bromoisobutyryl bromide (11.7 mL, 95 mmol) was dissolved in 50 mL dry DCM, and then was added gradually to the solution by an addition-funnel while the solution temperature was kept below 5 °C with the aid of an ice-cooled water bath over 1 h. The reaction mixture was stirred for another 48 h at room temperature. The formed salt was filtered off. The crude solution was washed twice with 1 M HCl_(aq)_, twice with saturated NaHCO_3_ solution, and twice with distilled water. The organic phase was dried with sodium sulfate. Then passed through silica column using EA/Hexane=1/6 as eluent. Pale yellow oil was obtained for 13.6 g (68%).

**Fig. 1:**
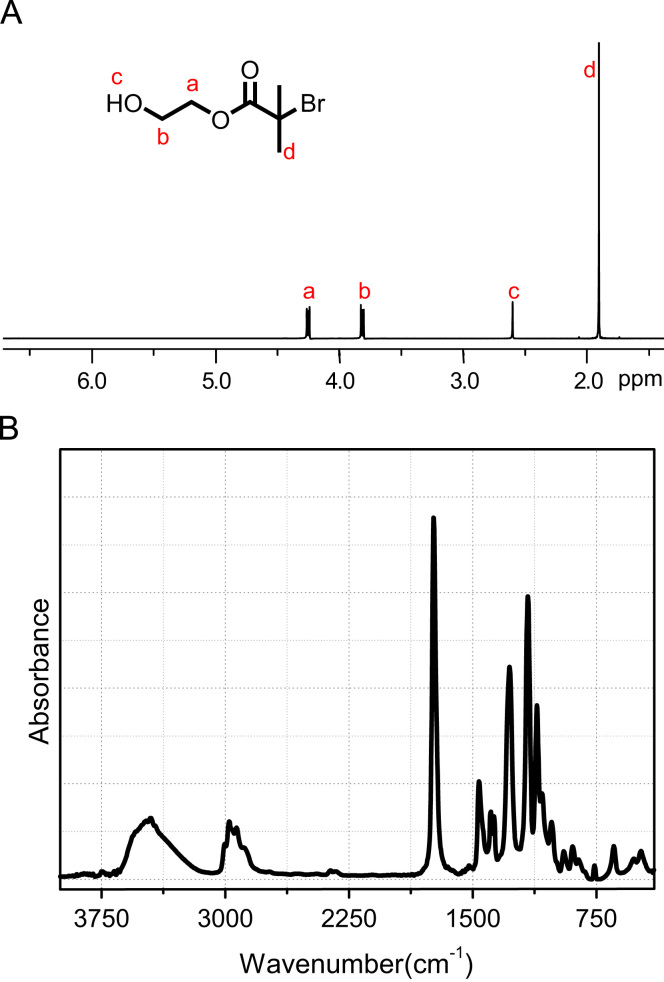
Characterization of HEBiB: spectra of (A) ^1^H NMR (400 MHz, CDCl_3_) and (B) FT-IR.

Characterization of HEBiB was shown in Fig. 1. In Fig. 1a, ^1^H NMR (400 MHz, CDCl_3_, *δ*=ppm): showed high purity and peak assigns were 1.90 (s, –C(CH_3_)_2_Br, 6H), 2.60 (s, –OH, 1H), 3.82 (m, –CH_2_–OH, 2H), and 4.25 (m, –CH_2_–CH_2_–, 2H). In Fig. 1b, FT-IR (KBr, cm^−1^) showed obvious absorbance peaks of 3350 (C—OH stretching), 2960 (CH stretching), 1730 (C=O stretching), 1463 (C—OH in-plane bending), 1270 (—COC— stretching), 1170, 1106 (—COC— bending), and 946 (C—OH out-of-plane bending). These results indicated HEBiB compound was successfully obtained in moderate yield and high purity.

### 1.5 SI ATRP from HEBiB/TOCN-Br (macro)initiators with St

A Schlenk flask was charged with St (4.80 mL, 32.0 mmol), HEBiB (15.0 μL, 0.106 mmol), *N*,*N*,*N*´,*N*´´,*N*´´-pentamethyldiethylenetriamine (PMDETA, 22.0 μL, 0.106 mmol), TOCN-Br (0.20 g), and anisole (4.30 mL) (St/HEBiB/PMDETA=300:1:1; [St]_0_=4.2 M) and capped with a rubber septum. The mixture was degassed through three freeze-pump-thaw cycles to remove O_2_ and backfilled with N_2_. CuBr (HEBiB/CuBr=1:1) was added to the frozen solution. The flask was closed, evacuated, and degassed through two additional freeze-pump-thaw cycles. At this stage (*t*=0), a sample was taken via syringe and then the flask was immersed in a thermostatted oil bath at 90 °C. During the polymerization process, aliquots were regularly removed to allow monitoring (through gas chromatography (GC), using anisole as an internal standard) of the St conversion. The polymerization was stopped by cooling the flask in an ice bath and exposing the contents to air. The resulting mixture was diluted with tetrahydrofuran (THF); the product was precipitated into MeOH, collected, and dried under vacuum. The free PSt homopolymer and residual catalyst were removed through a few cycles of Soxhlet extraction, using THF (24 h) and MeOH (24 h). After drying under vacuum, TOCN-*g*-PSt was obtained as a white powder.

**Fig. 2:**
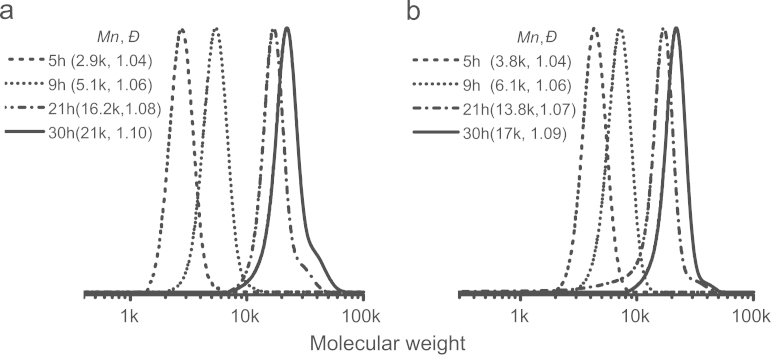
GPC traces of the products of surface0initiated atom transfer radical polymerization (SI ATRP) with styrene (St) in the presence of (a) TOCN-Br2 (Br atoms with 7.02 wt%) and (b) TOCN-Br3 (Br atoms with 8.70 wt%). Conditions were referred to Ref. [1, Figure 9] (TOCN-Br: TEMPO-oxidized cellulosed nanofibrils with bromoisobutyrate ATRP initiating site; *M*_n_: number-average molecular weight; *Đ*: molecular weight distribution).

In our previous study of Ln(*M*_0_/*M*) vs time plots [1], it showed gradual increases in conversion over time and linear first-order kinetics. In Fig. 2, GPC traces of the SI ATRP of St from two different TOCN-Br samples were recorded in various periods of reaction time. Clear changes in molecular weight (values of *M*_n_ increased to ca. 20 k) and narrow unimodal GPC peaks (*Đ*≤1.1) are clearly evident from the onset of the reactions. The influence of the content of immobilized initiating groups on the reaction kinetics was negligible. The results depicted a typical RDRP fashion, implying successful SI ATRPs could be achieved in the presence of TOCNs presenting neutral carboxylic acid sodium salt groups.

### 1.6 Contact angle measurements

**Fig. 3:**
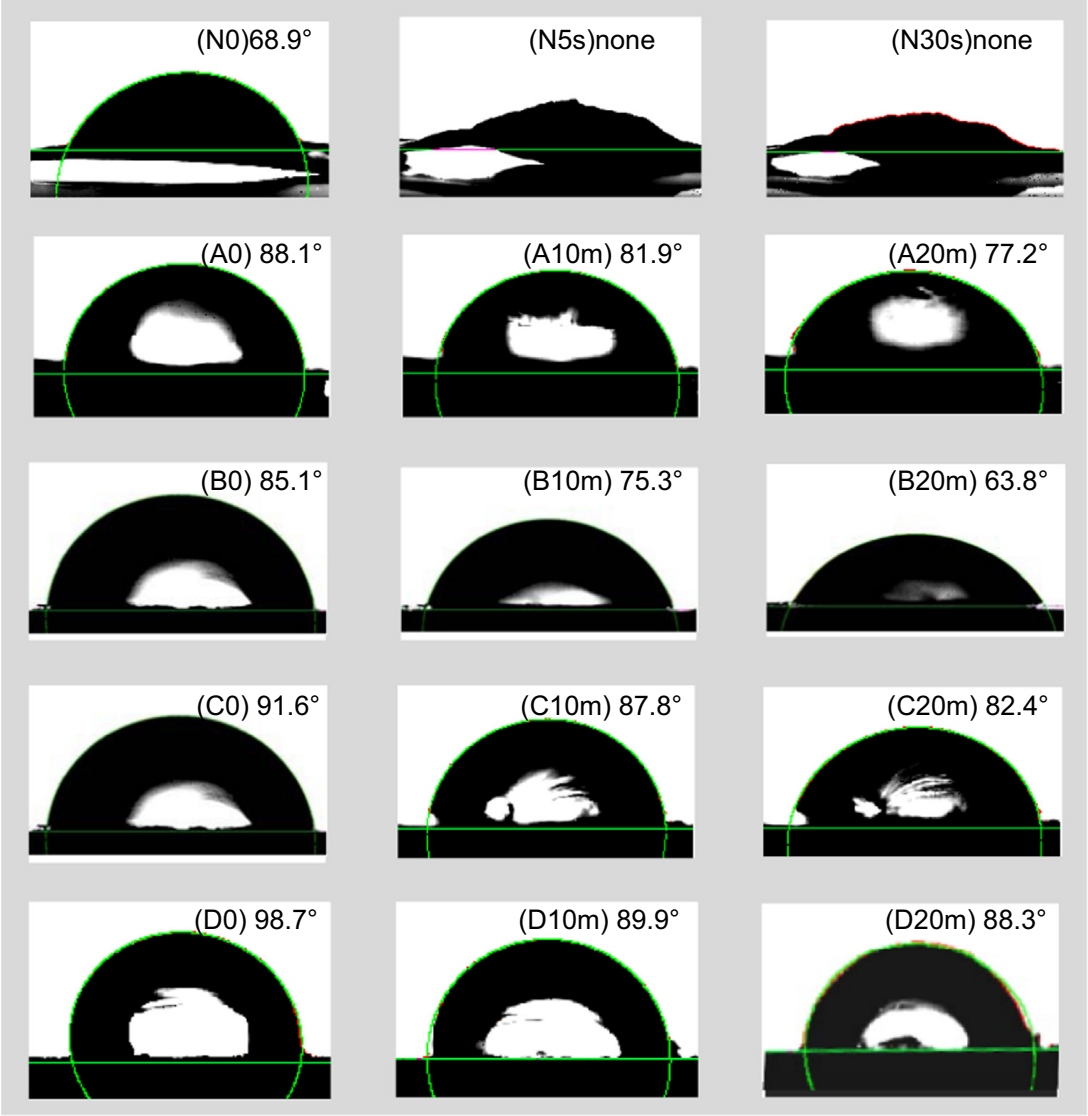
Images and CAs (deg) of (nano)celluloses after various static measuring times (N: TOCN; A–D: samples were from Ref. [1, Table 3]; s: sec; m: min).

Dried samples were pressed at ca. 50 psi to obtain flat platelets under ambient and dried in vacuum prior to measure CAs using a KRÜSS G10 instrument. As shown in Fig. 3, CA images of the various samples after various measuring times were displayed. Obvious reversibility between hydrophilicity and hydrophobicity in a certain period of time was observed. This reversibility has demonstrated its effectiveness on the adsorbing organic compounds and metal ions in aqueous media [1].
